# Charcot–Marie–Tooth type 2B disease-causing RAB7A mutant proteins show altered interaction with the neuronal intermediate filament peripherin

**DOI:** 10.1007/s00401-012-1063-8

**Published:** 2012-11-23

**Authors:** Laura Cogli, Cinzia Progida, Claire L. Thomas, Bradley Spencer-Dene, Claudia Donno, Giampietro Schiavo, Cecilia Bucci

**Affiliations:** 1 Department of Biological and Environmental Sciences and Technologies (DiSTeBA), University of Salento, Via Provinciale Monteroni 165, 73100 Lecce, Italy; 2 Molecular NeuroPathobiology, Cancer Research UK London Research Institute, 44 Lincoln’s Inn Fields, London, WC2A 3LY UK; 3 Experimental Pathology Laboratories, Cancer Research UK London Research Institute, 44 Lincoln’s Inn Fields, London, WC2A 3LY UK; 4 Present Address: Department of Molecular Biosciences, Centre for Immune Regulation, University of Oslo, 0316 Oslo, Norway

**Keywords:** RAB7, RAB7A, Peripherin, Rab proteins, Intermediate filaments, Charcot–Marie–Tooth, Two-hybrid

## Abstract

Charcot–Marie–Tooth type 2B (CMT2B) is a peripheral ulcero-mutilating neuropathy caused by four missense mutations in the *rab7a* gene. CMT2B is clinically characterized by prominent sensory loss, distal muscle weakness leading to muscle atrophy, high frequency of foot ulcers and infections that often results in toe amputations. RAB7A is a ubiquitous small GTPase, which controls transport to late endocytic compartments. Although the biochemical and functional properties of disease-causing RAB7A mutant proteins have been investigated, it is not yet clear how the disease originates. To understand how mutations in a ubiquitous protein specifically affect peripheral neurons, we performed a two-hybrid screen using a dorsal root ganglia cDNA library with the purpose of identifying RAB7A interactors specific for these cells. We identified peripherin, an intermediate filament protein expressed primarily in peripheral neurons, as a putative RAB7A interacting protein. The interaction was confirmed by co-immunoprecipitation and pull-down experiments, and established that the interaction is direct using recombinant proteins. Silencing or overexpression of wild type RAB7A changed the soluble/insoluble rate of peripherin indicating that RAB7A is important for peripherin organization and function. In addition, disease-causing RAB7A mutant proteins bind more strongly to peripherin and their expression causes a significant increase in the amount of soluble peripherin. Since peripherin plays a role not only in neurite outgrowth during development but also in axonal regeneration after injury, these data suggest that the altered interaction between disease-causing RAB7A mutants and peripherin could play an important role in CMT2B neuropathy.

## Introduction

Charcot–Marie–Tooth (CMT) disease is a group of genetically and clinically heterogeneous neuronal disorders [76, 79]. This neuropathy, also called hereditary motor and sensory neuropathy (HMSN), has a prevalence of 1:2,500 and its onset is usually within the first or second decade of life. CMT disease has a major impact on motor and sensory neurons and is characterized by progressive weakness of the distal muscles, first affecting lower legs and feet and then leading to muscle atrophy [6, 70, 81]. CMT type 2B (CMT2B) is an axonal autosomal dominant form of the neuropathy characterized by prominent distal sensory loss, muscle weakness and atrophy, and frequent ulcers leading to toe amputations, resulting in the alternative name of ulcero-mutilating neuropathy [1, 80]. Four missense mutations in the *rab7a* gene on chromosome 3q21 cause CMT2B [30, 50, 66, 88]. RAB proteins are small GTPases involved in the regulation of vesicular intracellular trafficking and signal transduction [17, 83]. Indeed, they participate in several steps of membrane traffic being involved in budding, moving, tethering and fusion of vesicles to the target compartment [83].

Two different forms of RAB7 have been identified. In addition to RAB7A, a close homologue that displays high sequence similarity to RAB7A has been identified and called RAB7B [96]. RAB7A and RAB7B have different functions: RAB7A is localized to late endosomes and controls transport to late endosomes and lysosomes as well as maturation of phagosomes and autophagosomes [19, 21, 28, 47, 54], while RAB7B controls transport from endosomes to the Golgi apparatus [73, 74]. The CMT2B-causing RAB7A mutant proteins have altered GDP/GTP cycle, resulting in an increased nucleotide dissociation rate constant (*K*_off_) for both GDP and GTP. As such, these mutants release bound nucleotides faster than the wild type (wt) protein and, consequently, they show also impaired GTPase activity per binding event [37, 63, 82]. In addition, these mutant proteins show a much higher *K*_off_ for GDP than for GTP and thus they are predominantly in the GTP-bound form in the cell and they bind more strongly to several RAB7A effectors [37, 63, 82].

Importantly, RAB7A controls axonal retrograde trafficking and signaling of the nerve growth factor (NGF) receptor TrkA [39, 78]. As a result, expression of the RAB7A T22 N dominant negative mutant causes a strong increase in neurite outgrowth [78]. Accordingly, CMT2B-causing RAB7A mutant proteins alter TrkA and Erk1/2 endosomal signaling in response to NGF, leading to a strong inhibition of neurite outgrowth in different cell lines [9, 34, 95]. Although these biochemical and functional data obtained with CMT-causing RAB7A mutants start to unravel the molecular mechanisms underlying CMT2B, it is not yet clear why expression of these mutant proteins specifically lead to a peripheral neuropathy [33].

In the present study, we decided to search for RAB7A interactors in dorsal root ganglia (DRG) cells to explain how mutations in a ubiquitous protein mainly affect peripheral neurons. Here, we report for the first time that RAB7A interacts directly with peripherin, a 57 kDa type III neuronal intermediate filament protein.

Intermediate filaments are a major component of the cytoskeleton, important for organelle positioning, transport and function [49]. Peripherin is primarily expressed in peripheral neurons and in discrete populations of CNS neurons that project towards peripheral structures [5, 13, 42, 59, 71, 72]. Peripherin is mutated in amyotrophic lateral sclerosis (ALS) and a neurotoxic splice variant has been identified in a mouse model of ALS [45, 60, 77, 94]. Also, an aggregate-inducing peripherin isoform is upregulated in ALS [94]. Importantly, peripherin is thought to be important in axonal regeneration [25, 26, 61, 67, 92]. Indeed, it has been suggested that the regeneration program is at least in part based on changes to intermediate filament composition and dynamics [87, 92]. In particular, peripherin is differentially regulated in regenerating and non-regenerating sensory neurons, and it plays a role during the elongation of motor axons [25, 75, 87, 92]. The abnormalities in the binding of peripherin to disease-causing RAB7A mutants described in this manuscript suggest that the interaction of this small GTPase to specific components of the neuronal cytoskeleton could be an important mechanism in the pathogenesis of CMT2B.

## Materials and methods

### Cells and reagents

All chemicals and cell culture reagents were purchased from Sigma-Aldrich (St. Louis, MO, USA). Restriction and modification enzymes were from New England Biolabs (Ipswich, MA, USA). HeLa (human cervix carcinoma), PC12 (rat pheochromocytoma) and Neuro2A (mouse neuroblastoma) cell lines were grown in DMEM supplemented with 10 % heat-inactivated fetal calf serum, 2 mM glutamine, 100 U/ml penicillin and 10 mg/ml streptomycin, in a 5 % CO_2_ incubator at 37 °C. For all experiments, only cells at passage number <20 were used.

### Neuronal cultures

DRG neurons were isolated from E13.5 mouse embryos using standard procedures [46]. Briefly, ganglia were isolated in Hank’s buffered salt solution (HBSS) and digested in collagenase IV (C5138; Sigma Aldrich, Dorset, UK)/dispase II (165 859; Roche Diagnostics Ltd. West Sussex, UK) for 40 min at 37 °C. The cell pellet was triturated with a fire-polished Pasteur pipette in 2 ml HBSS. A 5-ml solution of Percoll (17089102; GE Healthcare Life Sciences, Buckinghamshire, UK) in L-15 medium (1 ml Percoll mixed with 4 ml L-15) was then carefully added to the bottom of a Falcon tube. Dissociated DRG neurons were centrifuged through this Percoll cushion for 8 min at 380*g*. Cells were washed with 2 ml L-15 medium, re-centrifuged as before and then resuspended in a minimum volume of bare-bones medium [46] supplemented with 200 ng/ml NGF. DRG neurons were plated on poly-ornithine and laminin-coated coverslips and kept in complete medium in a 7.5 % CO_2_ humidified incubator at 37 °C for 5 days.

### Two-hybrid assay

5 μg of human DRG total RNA (CH636150; Takara Bio Europe/Clontech, Saint-Germain-en-Laye, France) was used to construct a two-hybrid cDNA library using a kit (CH630445; Clontech) following the manufacturer’s instructions. The library encodes proteins as C-terminal fusions with the GAL4 transcriptional activation domain in the pGADT7Rec vector.

A GAL4-binding domain/RAB7A ΔC fusion construct in the pGBKT7 vector encoding canine RAB7A was used to screen our custom-made DRG cDNA library using AH109 yeast cells [7, 8, 31]. Transformants were plated onto synthetic medium lacking His, Leu, and Trp and clones were picked up after 5–6 days at 30 °C. Specificity tests using the PGBKT7 vector, pGBKT7-RAB7A wt, pGBKT7-RAB7B wt or pGBKT7 peripherin, and PGADT7Rec-peripherin or PGADT7Rec-RAB7A wt constructs were performed. Clones were then assayed for growth on selective medium and for β-galactosidase activity using *o*-nitrophenyl-β-d-galactoside as a substrate as previously described [7, 8, 31].

### Plasmid construction

The RAB7A plasmids used in this study have been described previously [21, 35, 37, 82, 89]. MYC-tagged peripherin was obtained from Origene (RC207561). Peripherin ORF was transferred using *Asi*SI and *Mlu*I restriction enzymes to pCMV6-AN-HA (Origene, PS100013) to obtain a vector for the expression of HA-tagged peripherin. Peripherin ORF was amplified by PCR using the following oligonucleotides: 5′-ACGCGGATCCATGAGCCACCACCCGTCGG-3′ and 5′-CCGGAATTCTCAGTAACTGTGGGCAGAAG-3′ and cloned in the pGEX4T3 vector using *Bam*HI and *Eco*RI restriction sites to obtain a plasmid for the bacterial expression of GST-tagged peripherin. Peripherin 45 mutant was generated from peripherin cDNA using the following oligonucleotides: 5′-ATAGCGATCGCCATGGCCGAGGCCCTCAAC-3′ and 5′-CCGCGTACGCGTGTAACTGTGGGC-3′ and then cloned with *Asi*Si and *Mlu*I restriction enzymes into the myc-tagged vector. All constructs were sequence verified.

### Transfection and RNA interference

Transfection in Neuro2A and HeLa cells was performed using Metafectene Pro or Metafectene Easy from Biontex (Karlsruhe, D), following the manufacturer’s instructions. After 20 h, transfected cells were processed for immunofluorescence or biochemical assays. For RNA interference, siRNAs were purchased from MWG-Biotech (Ebersberg, D). We used the following oligonucleotides: siRNA mouse *rab7a*, sense sequence 5′-GGAUGACCUCUAGGAAGAATT-3′ and antisense sequence 5′-UUCUUCCUAGAGGUCAUCCTT-3′. As a negative control, we used a scrambled sequence: sense scrambled control 5′-ACUUCGAGCGUGCAUGGCUTT-3′ and antisense scrambled control 5′-AGCCAUGCACGCUCGAAGUTT-3′. Briefly, Neuro2A cells were plated 1 day before transfection in tissue culture dishes (6 cm diameter). Cells were transfected with siRNAs using Metafectene Si from Biontex (Karlsruhe, D) for 72 h and then processed for further assays.

### Co-immunoprecipitation and pull-down

For co-immunoprecipitation 25 μl of anti-HA or anti-MYC resin (Ezview Red Anti-HA or anti-Myc affinity gel from Sigma) was used according to the manufacturer’s instructions. Briefly, HeLa or Neuro2A cells were lysed with RIPA buffer (R078; Sigma-Alrich), centrifuged at 8,000 *g* to pellet nuclei, cellular debris and unbroken cells, and supernatants were incubated with the anti-HA or anti-MYC resin for 1 h at 4 °C on a rotating wheel. Immunoprecipitated samples were then loaded on SDS-PAGE and analyzed by western blotting. Co-immunoprecipitation of endogenous proteins was performed in Neuro2A cells using a crosslink immunoprecipitation kit (Pierce) following the manufacturer’s instructions. Briefly, mouse anti-RAB7 antibody (Sigma) or mouse IgG was crosslinked to a resin using disuccimidyl suberate (DSS) and incubated with pre-cleared Neuro2A lysate. After washing and elution immunoprecipitates were subjected to western blotting analysis using rabbit anti-RAB7A and anti-peripherin antibodies.

For pull-down His-tagged RAB7A wt and mutant proteins were expressed and purified from *E. coli* as previously described [29]. After purification, 20 μg of each purified protein was bound to NiNTA resin at 4 °C for 45 min. The resin was then washed three times for 5 min with a washing solution (NaH_2_PO_4_ 50 mM, NaCl 300 mM, imidazole 20 mM, pH 8.0) and incubated with Neuro2A cell extracts at 4 °C for 1 h. After washing, samples were loaded on SDS-PAGE and analyzed by western blotting.

For direct interaction GST, GST- and His-tagged proteins were expressed in bacteria and affinity purified as described [23, 29]. His-tagged RAB7A was incubated alone or in combination with GST or GST-tagged peripherin in PBS with 2 mM MgCl_2_ and GTP 0.8 mM for 1 h on a rotating wheel. Subsequently, pull-down was performed using a glutathione resin [40]. Samples were then subjected to SDS-PAGE and western blotting.

### Antibodies

Mouse monoclonal 9E10 anti-Myc (ab32), rabbit polyclonal anti-HA (ab9110) and rabbit polyclonal anti-peripherin 61 (ab4646) antibodies were from Abcam (Cambridge, UK), rabbit polyclonal anti-RAB7A (R4779, used at 1:500) and mouse monoclonal anti-α-tubulin (T5168) were from Sigma, while anti-peripherin antibodies (sc-7604) were from Santa Cruz Biotechnology (Santa Cruz, CA, USA). Primary antibodies were used at a 1:1,000 unless otherwise indicated. Secondary antibodies conjugated with fluorochromes or HRP were from Invitrogen (Milan, Italy) or Santa Cruz Biotechnology and used at 1:5,000 dilution.

### Western blotting

Total cell extracts were prepared by lysing the cells in 62.5 mM Tris–HCl, pH 6.8, containing 2 % (w/v) SDS and protease inhibitor cocktail. Differential extraction of solubile and filamentous peripherin (solubile and insolubile fractions) was performed as previously described [64]. Briefly, cells were harvested in low salt Triton-X100 buffer and incubated on ice for 30 min. Soluble and insoluble fractions were then separated by centrifugation. Fractions were loaded onto SDS-PAGE and separated proteins were transferred onto polyvinyl difluoride (PVDF) membranes from Millipore (Milan, Italy). The filter was blocked in 5 % milk in PBS for 30 min at room temperature, incubated with the appropriate antibody and then with a secondary antibody conjugated with HRP (diluted 1:5,000). Bands were visualized using enhanced chemiluminescence (GE, Milan, Italy) or western blotting Luminol Reagent (SantaCruz Biotechnology).

### Animal experiments

All animal experiments were carried out under license from the UK Home Office in accordance with the Animals (Scientific Procedures) Act 1986 and following approval from the Cancer Research UK Ethical Review Committee. Wt C57BL/6 mice were housed in a controlled temperature and humidity environment and maintained on a 12 h light/dark cycle with access to food and water provided ad libitum. Mice were terminally anaesthetized with sodium pentobarbitone and transcardially perfused with 4 % (w/v) paraformaldehyde in 0.1 M PBS.

### Immunofluorescence microscopy

Neuro2A cells grown on 12-mm round glass coverslips were permeabilized, fixed and incubated with primary and secondary antibodies as described previously [20, 22]. Images of Neuro2A cells represent maximum-intensity projections of Z stacks.

DRGs were fixed with 4 % paraformaldehyde (PFA) in PBS, permeabilized for 15 min with 0.1 % Triton X-100 in PBS, washed and blocked with 2 % bovine serum albumin (BSA) in PBS (blocking solution) for 1 h. Immunostaining with goat polyclonal anti-peripherin (sc7604, Santa Cruz; 1:100), rabbit monoclonal anti-RAB7A (9367S, Cell Signaling; 1:20) or mouse monoclonal anti-RAB7A (R8779, Sigma; 1:200) was carried out for 1 h in blocking solution followed by AlexaFluor555-conjugated anti-goat and AlexaFluor488-conjugated anti-rabbit secondary antibodies (30 min). All washes were in PBS except for final wash with water before mounting coverslips.

Sciatic nerves were removed, post-fixed overnight and then arranged in a longitudinal orientation in 2 % agarose before paraffin processing, embedding and sectioning at 4 µM onto charged slides. Sections were blocked with 10 % normal donkey serum/1 % BSA and stained overnight at 4 °C with rabbit monoclonal anti-RAB7A and goat polyclonal anti-peripherin. Following extensive washing, sections were stained with donkey secondary antibodies for 1 h at room temperature, washed and then mounted in Hardset Mount containing DAPI (Vector). The antibodies all required 15 min microwaving in 0.1 M citrate buffer pH 6.0 for antigen retrieval.

Samples were imaged by confocal microscopy with a Zeiss LSM 510 equipped with a 63×, 1.4 NA Plan Apochromat oil-immersion objective. Images were processed using Zeiss LSM 510 software.

### Quantification and statistical analysis

Protein levels were quantified by densitometry using the ImageJ software. The levels of peripherin were normalized against α-tubulin or against RAB7A, and analyzed statistically using the Student’s *t* test for unpaired data. The analysis was based on the comparison of cells expressing a HA-tagged RAB7A mutant protein and cells expressing HA-tagged RAB7A or control cells as indicated.

## Results

### Identification of peripherin as a RAB7A interacting protein

**Fig. 1:**
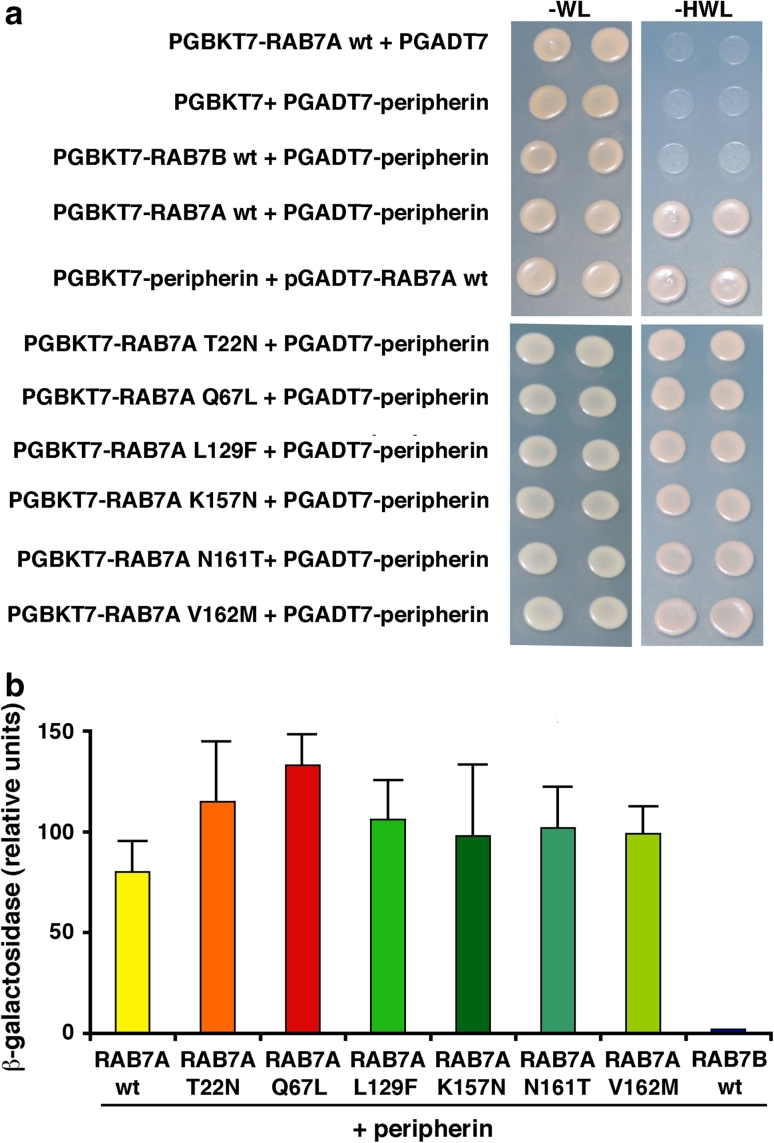
Specific interaction between RAB7A and peripherin in the two-hybrid system. **a** Yeast AH109 cells transformed with PGBKT7 vector, PGBKT7-*rab7b* wt or PGBKT7-*rab7a* wt or mutant constructs, and with pGADT7Rec vector, pGADT7Rec-peripherin or pGADT7Rec-RAB7A wt, as indicated, were plated on −WL and −HWL synthetic medium and incubated at 30 °C for 2 days. **b** The indicated double transformants of PGBKT7-*rab7b* wt, PGBKT7-*rab7a* wt or mutant constructs, and pGADT7Rec-peripherin were assayed for β-galactosidase liquid assay using ONPG as substrate in order to quantify the interaction. The *values* represent the mean of three independent experiments ± standard deviation

To identify novel RAB7A-interacting proteins specific for peripheral neurons, we decided to construct a human DRG cDNA library suitable for two-hybrid screens and use a Gal4-binding domain/RAB7A fusion construct as a bait. The final three amino acids of RAB7A (Cys-Ser-Cys) were deleted to prevent C-terminal prenylation as such modification might cause high background in the assay. From 1 × 10^6^ primary transformants and 27 putative positive clones, 11 were encoding putative true positives that did not activate transcription in the presence of a non-specific test bait. Five of the positive clones were encoding Rab Interacting Lysosomal Protein (RILP) and one Prenylated Rab Acceptor 1 (PRA1), two previously identified RAB7A interacting proteins [18, 23]. The remaining five clones contained part of the coding region and the 3′ untranslated region of peripherin cDNA, with the longest peripherin clone encoding from residue 280 to the end of the protein. The specificity of the interaction of RAB7A and peripherin was confirmed by the growth of yeast cells expressing RAB7A wt and peripherin on synthetic medium lacking leucine, tryptophan and histidine (Fig. 1a). In contrast, yeast cells expressing peripherin alone or together with RAB7B were unable to grow without histidine (Fig. 1a). The specificity of this interaction was also confirmed using a quantitative β-galactosidase assay (Fig. 1b). Importantly, we tested in this system the ability of peripherin to interact with the different RAB7A mutant proteins: the dominant negative RAB7A T22 N, the constitutively active RAB7A Q67L and the CMT2B-causing mutants RAB7A L129F, RAB7A K157 N, RAB7A N161T and RAB7A V162 M. As shown in Fig. 1, all RAB7A mutant proteins efficiently interact with peripherin.

### CMT2B-causing RAB7A mutant proteins show altered interaction with peripherin

**Fig. 2:**
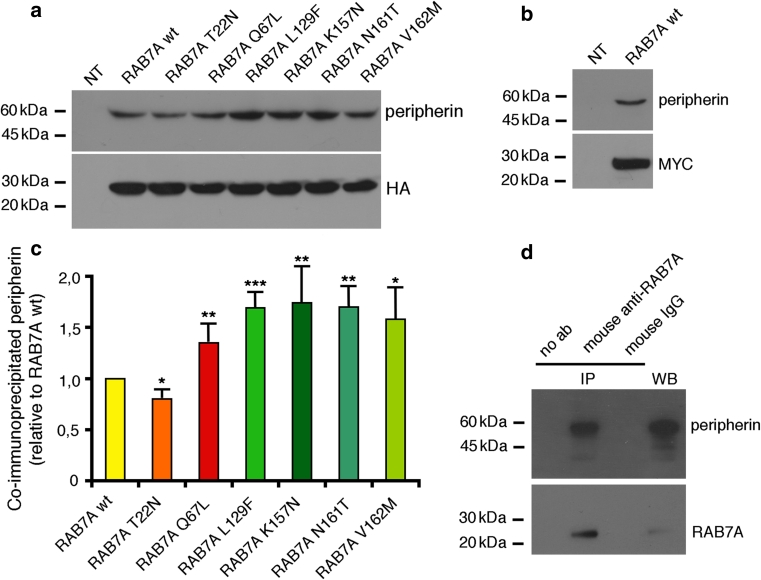
RAB7A co-immunoprecipitates with peripherin. **a** HA-tagged RAB7A wt and mutant proteins were expressed in Neuro2A cells, as indicated, immunoprecipitated with an anti-HA antibody and subjected to western blot analysis using anti-peripherin and anti-HA antibodies. **b** Immunoprecipitates obtained from Neuro2A cells expressing MYC-tagged RAB7A wt using an anti-MYC antibody were subjected to western blot analysis with anti-peripherin and anti-MYC antibodies. **c** Quantification of immunoprecipitated peripherin. *Values* are the mean of three independent experiments ± standard deviation. The intensities were quantified by densitometry, normalized against the amount of the RAB7A wt or mutant protein present, and plotted as a percentage of the intensities obtained using RAB7A wt. Data were analyzed statistically using unpaired Student’s *t* test. The values of cells expressing RAB7A mutant proteins were found to be significantly different from the values of cells expressing RAB7A wt (*p < 0.05, **p < 0.01, ***p < 0.001). **d** Total extracts of Neuro2A cells and immunoprecipitates obtained with no antibodies (no ab), with mouse anti-RAB7A and with mouse IgG were subjected to WB analysis using rabbit anti-RAB7A and rabbit anti-peripherin antibodies

To confirm the results obtained with the two-hybrid screening, we investigated whether RAB7A and peripherin co-immunoprecipitate in Neuro2A cells. This cell line and PC12 cells are ideal cellular models to study the RAB7A-peripherin interaction since they both express endogenous peripherin [2, 65]. Neuro2A cells were transfected with HA-tagged or MYC-tagged RAB7A wt and immunoprecipitated using anti-HA or anti-MYC beads. Both HA-tagged (Fig. 2a) and MYC-tagged (Fig. 2b) RAB7A immunoprecipitates contained peripherin, thus confirming that the two proteins exist in a complex in Neuro2A cells. Interestingly, the RAB7A Q67L constitutively active mutant and the disease-causing mutant proteins RAB7A L129F, RAB7A K157 N, RAB7A N161T and RAB7A V162 M were all able to co-immunoprecipitate peripherin more efficiently then RAB7A wt, thus demonstrating that they interact more strongly with peripherin (Fig. 2a). Indeed, quantification of three independent experiments indicates that disease-causing RAB7A mutant proteins are able to co-immunoprecipitate peripherin about 60 % more efficiently than RAB7A wt (Fig. 2c). Similar results were obtained in PC12 cells (data not shown). Strikingly, this binding was also shown using endogenous proteins (Fig. 2d), suggesting that the interaction between RAB7A and peripherin is of physiological significance.

**Fig. 3:**
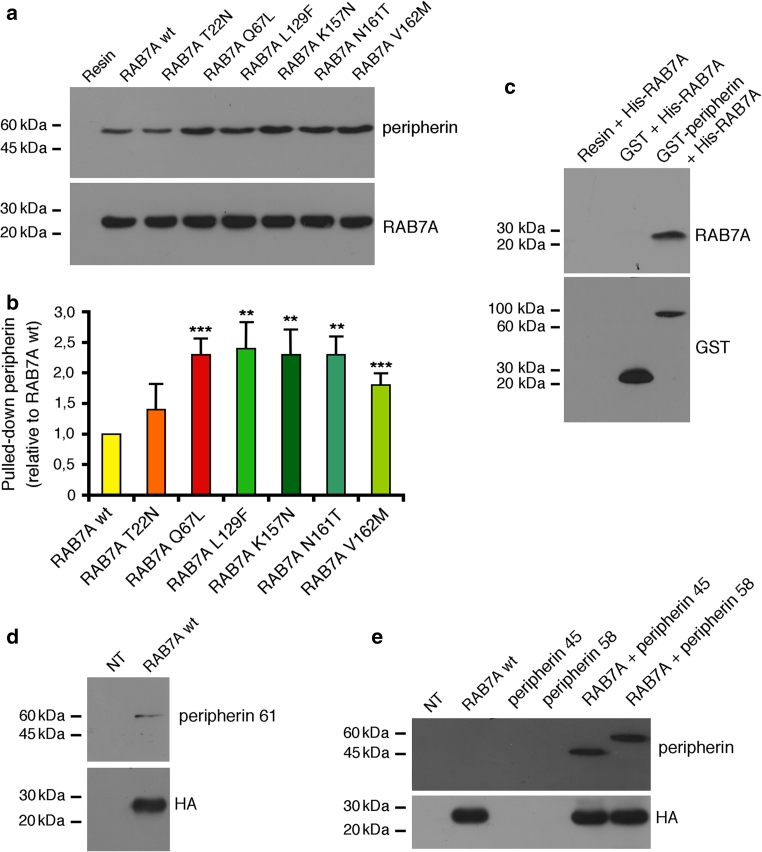
RAB7A interacts with peripherin and with peripherin’s isoforms 45 and 61. **a** Bacterially expressed and affinity purified His-tagged RAB7A wt and mutant proteins were incubated with total extract of Neuro2A cells, purified with NiNTA resin and subjected to western blot analysis using anti-RAB7A and anti-peripherin antibodies, as indicated. **b** Quantification of pulled down peripherin. The *values* represent the mean of four independent experiments ± standard deviation. The intensities were quantified by densitometry, normalized against the amount of the RAB7A wt or mutant protein present and plotted as a percentage of the intensities obtained using RAB7A wt. Values obtained using disease-causing RAB7A mutant proteins were found to be significantly different form the values obtained with RAB7A wt (*p < 0.05, **p < 0.01, ***p < 0.001). **c** Bacterially expressed GST or GST-peripherin immobilized on a glutathione resin, or a glutathione resin alone were incubated with purified His-RAB7A. Samples were then subjected to western blot analysis using anti-RAB7A and anti-GST antibodies. **d** Immunoprecipitates obtained from total extracts of Neuro2A cells overexpressing HA-RAB7A wt using anti-HA antibodies were subjected to western blot analysis using anti-HA and anti-peripherin 61 antibodies. **e** Total extracts of control HeLa cells (NT) or of cells expressing HA-RAB7A wt, MYC-peripherin 45 and/or MYC-peripherin 58, as indicated, were subjected to immunoprecipitation using anti-HA antibody and then to western blot analysis using anti-peripherin and anti-HA antibodies

We then incubated recombinant His-RAB7A with Neuro2A extracts. His-RAB7A wt was able to pull-down peripherin, thus providing an independent confirmation of the interaction between these two proteins (Fig. 3a). Importantly, pull-down was more efficient when the constitutively active RAB7A Q67L mutant or the CMT2B RAB7A mutants were used (Fig. 3a). Quantification revealed that these mutant proteins were able to pull-down twofold levels of peripherin compared to RAB7A wt (Fig. 3b).

Finally, we used pull-downs of recombinant purified proteins to test whether the interaction between RAB7A and peripherin is direct. To this end, bacterially expressed GST or GST-tagged peripherin was incubated with purified His-RAB7A and then subjected to GST pull-down using a glutathione resin. The resin was then extensively washed and bound proteins were analyzed by western blot. Strikingly, anti-RAB7A antibodies recognized a band corresponding to His-RAB7A only in the GST-peripherin sample (Fig. 3c). Altogether these data indicate that RAB7A interacts directly with peripherin and that CMT2B-causing RAB7A mutant proteins bind peripherin more efficiently than RAB7A wt.

In addition to the main form of peripherin with an apparent molecular weight of 58 kDa (also called Per-58), other isoforms of peripherin derived from alternative splicing and/or intron retention have been identified in mammalian cells, such as Per-61, Per-56, Per-45 and Per-28 [64, 65]; novel forms may also be present [64, 65, 77, 94]. Interestingly, the binding of RAB7A is not restricted to Per-58 since RAB7A was shown to interact with endogenous Per-61 in Neuro2A cells, using a specific antibody for this isoform, and with overexpressed Per-45 in HeLa cells (Fig. 3d, e).

### Intracellular localization of RAB7A and peripherin

**Fig. 4:**
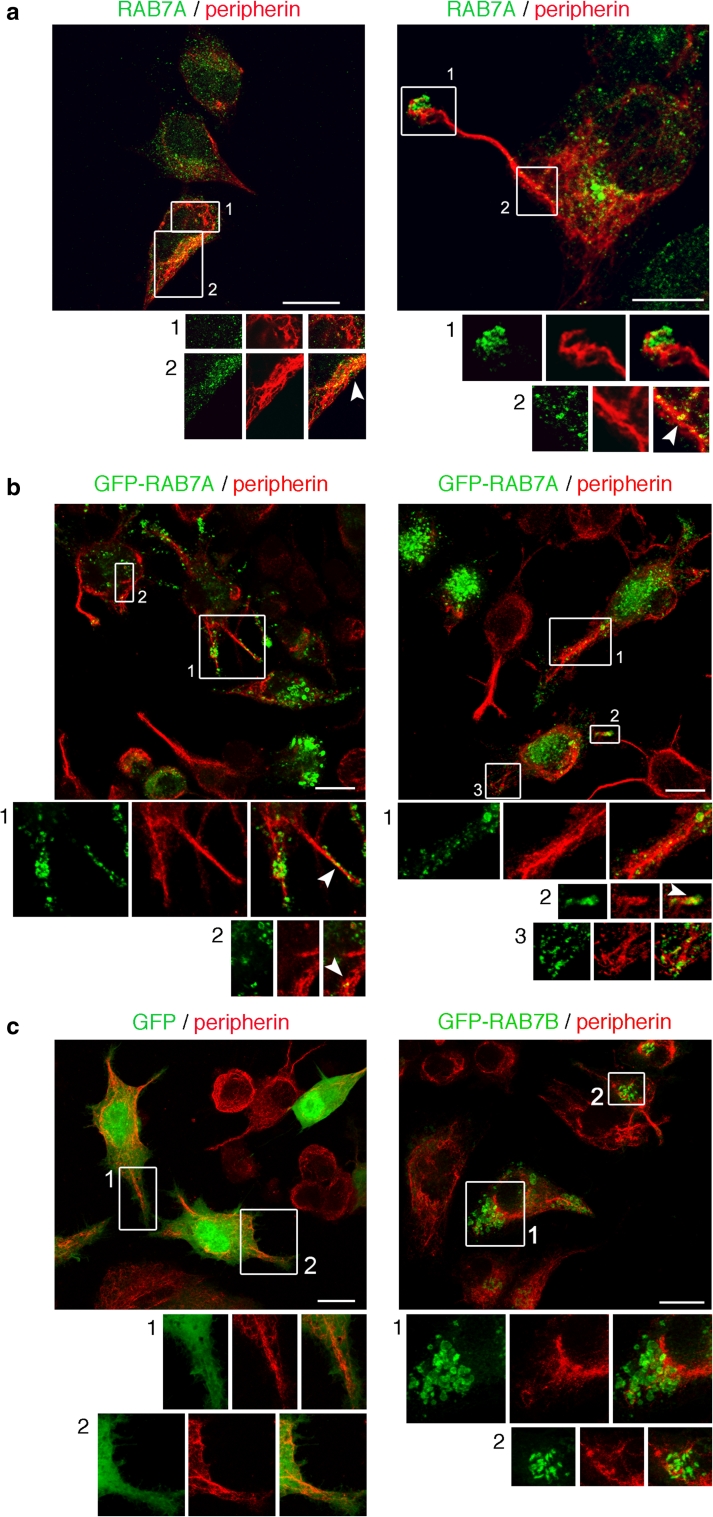
Immunofluorescence analysis of intracellular localization of RAB7A wt and peripherin. **a** Neuro2A cells were subjected to immunofluorescence using anti-RAB7A and anti-peripherin antibodies followed by secondary antibodies conjugated with Cy2 (*green*) or Cy3 (*red*), respectively. **b**, **c** Neuro2A cells expressing GFP, GFP-RAB7B, or GFP-RAB7A, as indicated, were subjected to immunofluorescence analysis using anti-peripherin antibodies followed by a Cy3 (*red*)-conjugated secondary antibody. *Images* represent maximum-intensity projections of Z stacks. For each image, magnifications of the *boxed areas* are shown in the respective *lower insets*. *Arrowheads* point to RAB7A-bearing vesicles juxtaposed to peripherin filaments. *Scale bars* 10 μm

We then investigated the intracellular localization of RAB7A and peripherin in Neuro2A cell using confocal immunofluorescence analysis (Fig. 4). As expected, anti-RAB7A antibodies labeled vesicles and organelles, often concentrated in the perinuclear region, while the anti-peripherin stained a filamentous network that was also localized in cell protrusions (Fig. 4a). Interestingly, RAB7A-bearing vesicles were sometimes seen colocalizing with peripherin filaments (Fig. 4a, insets, arrowheads). To confirm these findings, we performed confocal immunofluorescence analysis using anti-peripherin antibodies on Neuro2A cells overexpressing GFP, GFP-RAB7A wt and GFP-RAB7B wt (Fig. 4b, c). As previously reported, GFP-tagged RAB7A wt was present on cytosolic vesicles and its overexpression caused enlargement and clustering of these vesicles in the perinuclear region (Fig. 4b) [21]. This analysis revealed a very limited but consistent co-localization of RAB7A with peripherin. In particular, some RAB7A-positive vesicles were seen to lie over peripherin filaments (Fig. 4b, insets, arrowheads). No similar close association of vesicles with peripherin filaments was observed in cells overexpressing GFP or GFP-RAB7B (Fig. 4c).

**Fig. 5:**
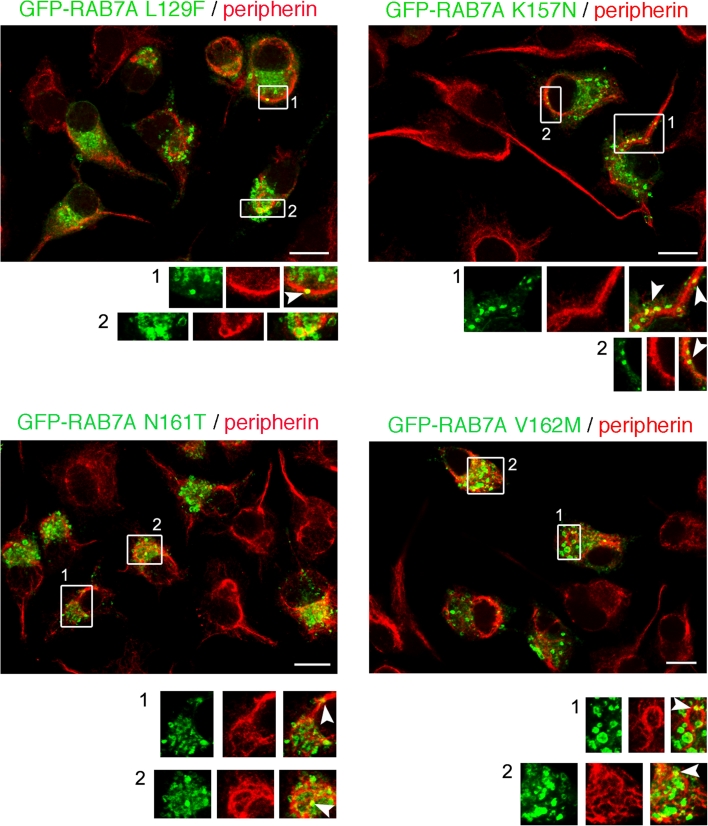
Immunofluorescence analysis of intracellular localization of CMT2B-causing Rab7 mutant proteins and peripherin. Neuro2A cells overexpressing GFP-RAB7A mutant proteins (*green*), as indicated, were subjected to immunofluorescence analysis using an anti-peripherin antibody followed by a Cy3 secondary antibody (*red*). *Images* represent maximum-intensity projections of Z stacks. For each image, magnification of the *boxed areas* is shown in the respective *lower insets*. *Arrowheads* point to RAB7A mutants-positive vesicles colocalizing to peripherin filaments. *Scale bars* 10 μm

A similar pattern was also detected when CMT2B-causative RAB7A mutant proteins were expressed (Fig. 5). Indeed, vesicle-bearing RAB7A mutants were found in close proximity to peripherin filaments (Fig. 5, insets, arrowheads).

**Fig. 6:**
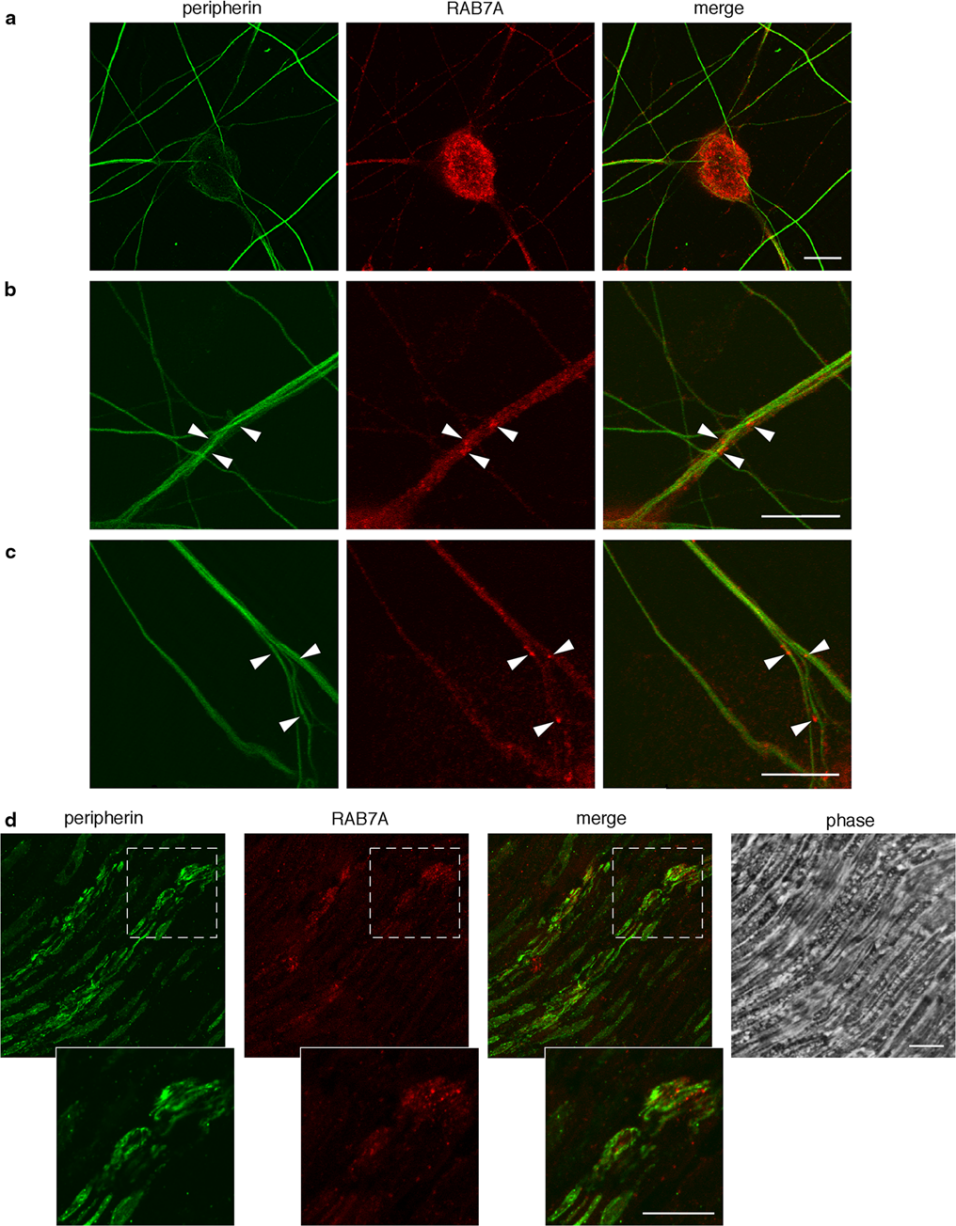
RAB7A and peripherin are co-expressed in DRGs and in the sciatic nerve. **a** Peripherin displays a filamentous distribution in the axonal network of embryonic DRGs cultured in vitro, which overlaps with RAB7A staining. **b**, **c** At close inspection, Rab7-positive organelles are often in close apposition to peripherin filaments (*arrowheads* point to RAB7-positive vesicles which overlap to peripherin staining). Similar results were obtained with two different RAB7A antibodies and are confirmed by the staining of Rab7 and peripherin in mouse sciatic nerve (**d**). **d** Both RAB7A and peripherin are co-expressed in a subset of nerve fibers, where RAB7A-positive organelles juxtapose to peripherin filaments. *Scale bars* 10 μm

We also tested the colocalization of endogenous peripherin and RAB7 in embryonic DRGs neurons, a well-established model of sensory neurons. As shown in Fig. 6, peripherin displays an extensive staining of the neuronal network, which is expected from its established role in the neuronal cytoskeleton [32]. In contrast, RAB7A antibodies label punctate structures widely distributed in the neuronal network and in the cell body (Fig. 6a), which are often juxtaposed to peripherin filaments (Fig. 6b, c). This partial co-distribution is also observed in sections of the sciatic nerve (Fig. 6d), confirming that RAB7A-positive organelles and peripherin are likely to be in close contact both in vitro and in vivo.

### RAB7A regulates peripherin assembly

**Fig. 7:**
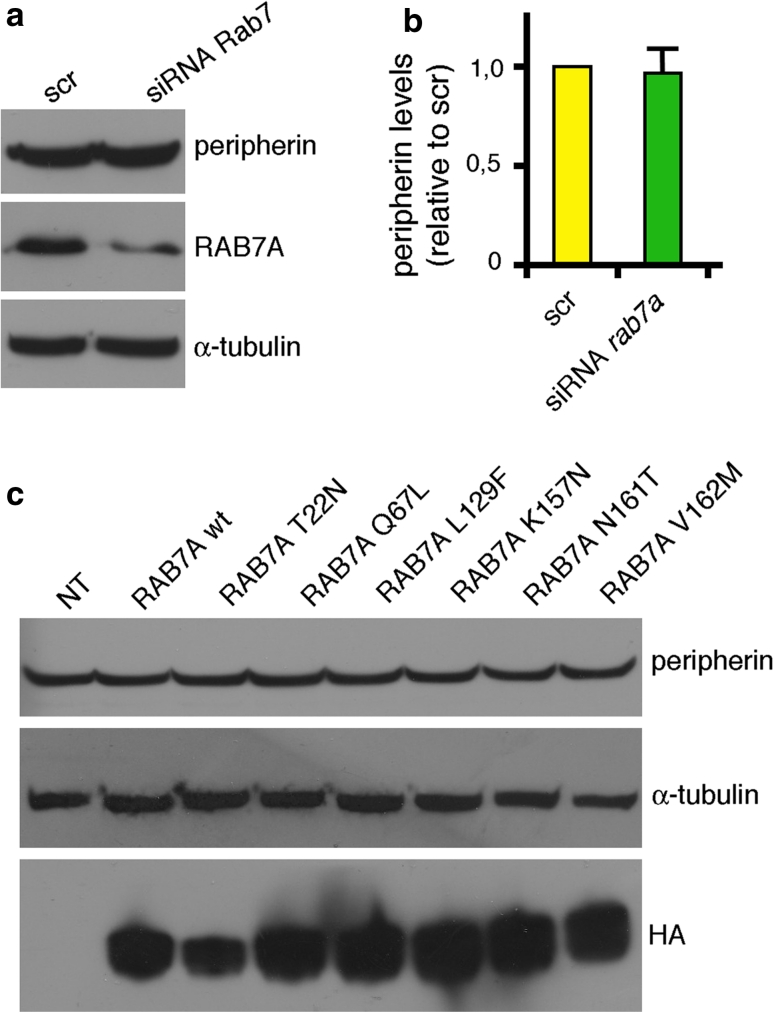
Altered expression of RAB7A does not alter peripherin levels. **a** Neuro2A cells were treated with control scrambled RNA (scr) or with *rab7a* siRNA as indicated. Total extracts were subjected to western blot analysis using anti-RAB7A and anti-peripherin antibodies. **b** Quantification of peripherin in *rab7a*-silenced cells. The *values* represent the mean of six independent experiments ± standard error. The intensities were quantified by densitometry, normalized against the amount of α-tubulin and plotted as a percentage of the intensities obtained in cells transfected with control RNA (scr). **c** Total extracts of Neuro2A control cells or cells overexpressing RAB7A wt and mutant proteins, as indicated, were subjected to western blot analysis using anti-peripherin and anti-HA antibody

**Fig. 8:**
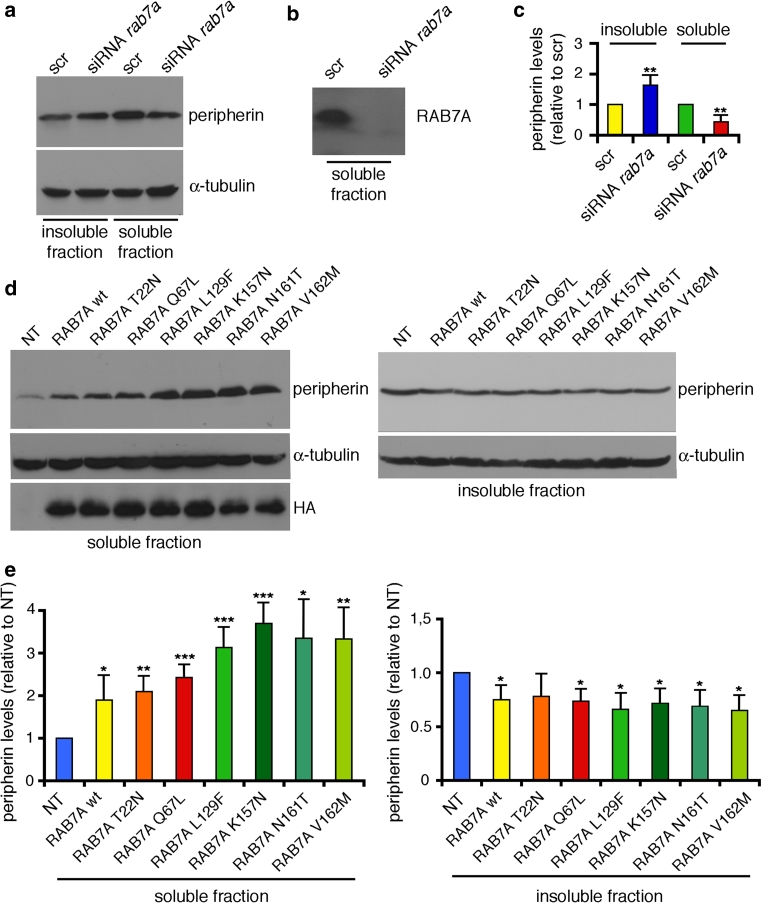
RAB7A regulates soluble/insoluble ratio of peripherin. **a**, **b** Neuro2A cells were treated with control scrambled RNA (scr) or with *rab7a* siRNA as indicated. Soluble and insoluble fractions were separated as described in “Materials and methods”, loaded on SDS-PAGE and subjected to western blot analysis using anti-peripherin (**a**), anti-α-tubulin (**a**) and anti-RAB7A (**b**) antibodies. **c** Quantification of peripherin in *rab7a*-silenced cells. The *values* represent the mean of three independent experiments ± standard deviation. The intensities were quantified by densitometry, normalized against the amount of α-tubulin and plotted as a percentage of the intensities obtained in cells transfected with control RNA (scr). **d** Neuro2A control cells (NT) or expressing HA-tagged RAB7A wt and mutant proteins as indicated were lysed. Soluble and insoluble fractions were collected from each sample, loaded on SDS-PAGE and subjected to western blot analysis using anti-peripherin, anti-α-tubulin and anti-HA antibodies. **e** Quantification of soluble and insoluble peripherin. The *values* represent the mean of four independent experiments ± standard deviation. The intensities were quantified by densitometry, normalized against the amount of α-tubulin and plotted as a percentage of the intensities obtained in control cells (cells transfected with an empty vector). Values obtained expressing disease-causing RAB7A mutant proteins were found to be significantly different from the values obtained in control cells (*p < 0.05, **p < 0.01, ***p < 0.001)

To start to understand the functional meaning of this interaction, we first decided to investigate whether RAB7A could alter the total amount of peripherin. RAB7A knock-down using RNA interference reduced *rab7a* mRNA levels to less than 20 % (data not shown) and endogenous RAB7A protein was no longer detectable by western blot (Figs. 7, 8). However, the total amount of peripherin in RAB7A-silenced Neuro2A cells was not changed significantly (Fig. 7a). Consistently, overexpression of HA-tagged RAB7A wt or mutants did not alter the total amount of peripherin in these cells (Fig. 7b).

Intermediate filaments are insoluble protein polymers that are assembled from soluble precursors [41, 49]. Intermediate filament proteins self-assemble to form coiled-coil dimers that associate laterally to form tetrameric complexes. Monomers, dimers and tetramers are entirely soluble. Tetramers associate laterally to form short filaments (also known as unit-length filaments) that longitudinally anneal to assemble long filaments that are highly insoluble under physiological conditions [41, 49]. Peripherin is assembled from soluble precursors in a similar way [44], thus soluble peripherin precursors could be separated from the insoluble peripherin filaments, and the peripherin soluble/insoluble ratio determined.

To check whether RAB7A influences peripherin organization, we decided to monitor the amount of peripherin present in the soluble and insoluble fractions in RAB7A-depleted Neuro2A cells. Interestingly, *rab7*a silencing caused a redistribution of peripherin with a decrease of the amount of peripherin in the soluble fraction and an increase in the insoluble fraction (Fig. 8a, b) as quantified in Fig. 8c. Consistently, expression of HA-tagged RAB7A wt caused a strong increase in the amount of soluble peripherin and a decrease in the amount of insoluble peripherin (Fig. 8d, e). Importantly, a stronger increase of soluble peripherin was observed upon expression of the HA-tagged RAB7A Q67L constitutively active mutant and the increase was even stronger upon expression of CMT2B-inducing RAB7A mutants (Fig. 8d). Quantification of four independent experiments indicated that upon expression of CMT2B-causing RAB7A mutants, soluble peripherin increased about threefold (Fig. 8e). In line with these results the presence of peripherin in the insoluble fraction diminished (Fig. 8e). Similar data were obtained in PC12 cells (data not shown).

## Discussion

Our results demonstrate a novel direct interaction between the small GTPase RAB7A and peripherin, a component of the intermediate filament cytoskeleton. Rab proteins act as master regulators of intracellular membrane traffic. Vesicle formation, their movement on cytoskeletal tracks, tethering, recognition and fusion to the target compartment are processes regulated by one or more of these small GTPases [83]. Intermediate filaments have also been directly connected with the regulation of membrane trafficking, as they are involved in organelle positioning and function [27, 44, 68, 84]. Indeed, intermediate filaments interact with several types of organelles, such as the nucleus, mitochondria, the Golgi apparatus, endosomes and lysosomes. In particular, neurofilaments are intimately connected with mitochondria and disruption of these connections alters mitochondrial morphology, distribution and functionality [24, 90]. Several intermediate filament proteins, such as vimentin, peripherin and α-internexin, are associated with the endosomal adaptor complex AP3 that mediates trafficking of cargo proteins to lysosome-related organelles [85]. Changes in the intermediate filament network alter not only the intracellular distribution of AP3 but also the positioning and functionality of late endosomes and lysosomes demonstrating a strong link between intermediate filaments and the endocytic pathway [85]. Our novel findings reporting the interaction between RAB7A and peripherin confirm the close functional relationship between intermediate filaments and regulators of endocytic trafficking. Moreover, our observation that RAB7A regulates peripherin soluble/insoluble ratio (Fig. 8) corroborates this hypothesis suggesting, for the first time, that RAB proteins could also direct vesicular trafficking by controlling intermediate filament dynamics.

Peripherin is predominantly expressed in the peripheral nervous systems and also in CNS neurons projecting towards peripheral structures [5, 13, 42, 59, 71, 72]. In particular, peripherin expression has been demonstrated in cranial nerves and spinal cord neurons, including motor neurons. Indeed, peripherin is expressed in cholinergic laterodorsal tegmentum (LDT) and pedunculopontine tegmentum (PPT) nuclei as well as in tuberomammellary neurons of the posterior hypothalamus [5, 13, 42, 59, 71, 72]. The presence of RAB7A effector protein expressed only in certain kinds of neurons and, in particular, in neurons of the peripheral nervous system might explain why mutations in a ubiquitous protein such as RAB7A cause a specific peripheral neuropathy. As a consequence, these considerations are likely to prompt searches for specific neuronal effectors of other ubiquitous proteins, whose mutations specifically affect the nervous system.

Peripherin is a neuronal differentiation marker induced by stimulation with NGF that has been suggested to have a role in neuritogenesis, axonal outgrowth and axonal regeneration [2, 4, 11, 43, 48, 56, 86]. Importantly, overexpression of peripherin causes degeneration of motor axons during aging inducing neuron dysfunction and the slowing down of neurofilament protein transport [10, 58, 67]. Peripherin overexpression also induces aberrant intermediate filament phosphorylation and the formation of neuronal intermediate filament aggregates, which cause swelling of ER and mitochondria [58]. Interestingly, peripherin is associated with pathological aggregates in patients with ALS and rare mutations in peripherin were identified in some sporadic ALS cases [36, 94]. Moreover, in a mouse model of ALS, a splice variant of peripherin seems to contribute to neurodegeneration [77]. Based on the data suggesting a role of peripherin in axonal outgrowth and our findings discussed above, we propose that the binding of peripherin to CMT2B-causing RAB7A mutants alters peripherin functions thus affecting neurofilament dynamics and as a consequence neurite outgrowth and axonal regeneration. This is particularly important since the possibility that CMT2B is caused by defects of axonal regeneration has not been yet explored. In addition, the exact role(s) of peripherin in neuronal differentiation and homeostasis is still unclear and what must also be considered is that peripherin-null mice failed to show strong phenotypes [42, 57].

The fact that RAB7A could regulate peripherin assembly and thus peripherin functions reveals a new mechanism that may potentially explain the peripheral neuropathy caused by RAB7A mutations. Indeed, we have demonstrated that CMT2B-causative RAB7A mutants bind more strongly to peripherin compared to RAB7A wt (Figs. 2, 3). Although their expression does not change the total levels of peripherin (Fig. 7), CMT2B-causative RAB7A mutants alter the peripherin soluble/insoluble ratio (Fig. 8), suggesting that the process of peripherin filament assembly is at least in part compromised. Peripherin and neurofilament triplet proteins co-assemble and are functionally interdependent [97]. Notably, peripherin mutations disrupt the neurofilament network while deletion of the neurofilament light gene leads to a reduction of peripherin in mouse sciatic nerve [45, 97]. In view of these data it is interesting to note that other forms of CMT disease are due to mutations of the neurofilament light gene and these mutations disrupt neurofilament assembly and axonal transport [14, 16]. Thus, defects in peripherin assembly caused by CMT2B-causing RAB7A mutant proteins could, in turn, affect neurofilament assembly and axonal transport leading to neurodegeneration. Future experiments using cultured neurons and animal models bearing mutations in RAB7A mimicking those found in CMT2B patients will be instrumental to test this hypothesis.

How may RAB7A affect peripherin assembly? One interesting possibility is that RAB7A sequesters peripherin precursors either in the cytoplasm or on endosomal membranes. This process could therefore sequester peripherin monomers, which would not be available for neurofilament assembly. Thus, if peripherin is involved in axonal regeneration, as previously suggested [25, 26, 61, 67, 92], the presence of CMT2B-causing mutants could be highly detrimental. Another possibility is that RAB7A binding could alter peripherin phosphorylation, either directly or indirectly. In fact, as with other intermediate filament proteins, peripherin is structured as a central coiled-coil α-helical rod domain flanked by a head and a tail domain. Head and tail domains of intermediate filament proteins are phosphorylated at several Ser/Thr sites and this has also been demonstrated for peripherin [3, 51, 55, 59]. Phosphorylation at certain sites has been shown to induce filament disassembly [53]. Interestingly, peripherin is a substrate for Akt [55], a kinase that is known to play a key role in many fundamental cellular processes, such as cell proliferation and differentiation. In neurons, Akt is also involved in neuronal survival and axonal formation, and it appears to have a crucial role in neuronal protection and axonal regeneration in peripheral neurons [15, 62, 69]. Whereas RAB5A attenuates Akt signaling through the recruitment of downstream effectors, RAB7A contributes to the maintenance of Akt survival signaling [12, 91]. It is thus tempting to speculate that Akt signaling could influence peripherin function and that RAB7A-dependent modulation of Akt activity may change the profile of peripherin phosphorylation, thus influencing peripherin function in axonal homeostasis. Alternatively, RAB7A mutants could indirectly affect peripherin phosphorylation by recruiting specific cytosolic kinases by virtue of their constitutively activated GTP-bound state, or by transiently localizing peripherin monomers/tetramers on endocytic membranes loaded with active kinases. In this regard, active kinase complexes as well as RAB7A have been found on signaling endosomes [38, 39, 52, 93], which may become sites of peripherin post-translational modification in CMT2B mutant neurons.

Further work will be necessary to define the physiological importance of the interaction between RAB7A and peripherin in vivo, and its relevance to peripheral neurons expressing CMT2B-causing RAB7A mutants. Thus, experiments on CMT2B cells and/or tissues are in progress and will be fundamental to unravel molecular basis of CMT2B peripheral neuropathy.
